# The association between serum magnesium and chronic kidney disease in Chinese adults: a cross-sectional study

**DOI:** 10.1186/s12889-023-17615-2

**Published:** 2024-01-15

**Authors:** Jing-Zhi Xie, Yuanyuan Huang, Xiao-Feng Zheng, Ruimei Feng, Xiao-Yun Li, Zi-Gui Zheng, Bing-Jing Jiang, Shanshan Du, Heng-Gui Chen, Yanfang Xu

**Affiliations:** 1 Department of Nephrology, Blood Purification Research Center, First Affiliated Hospital, Fujian Clinical Research Center for Metabolic Chronic Kidney Disease, Fujian Medical University, Chazhong Road 20, 350005 Fuzhou, Fujian Province China; 2 Department of Nephrology, Binhai Campus of the First Affiliated Hospital, National Regional Medical Center, Fujian Medical University, 350212 Fuzhou, China; 3 Department of Public Health, Fujian Normal University Hospital, Fujian Normal University, Fuzhou, Fujian Province China; 4 Department of Epidemiology and Health Statistics, School of Public Health, Fujian Medical University, Fuzhou, Fujian Province China; 5 Department of Preventive Medicine, School of Public Health, Fujian Medical University, No. 1 Xuefu North Rd, 350122 Fuzhou, Fujian Province China

**Keywords:** Serum magnesium, CKD, Chinese adults, Cross-sectional study

## Abstract

**Background:**

Magnesium (Mg) is both an essential macro-element and a known catalyst, and it plays a vital role in various physiological activities and mechanisms in relation to chronic kidney disease (CKD). However, epidemiological evidence involving this is limited and not entirely consistent. This study aims to explore the association of serum Mg concentrations with the risk of CKD among general Chinese adults.

**Methods:**

A total of 8,277 Chinese adults were included in the wave of 2009 from the China Health and Nutrition Survey (CHNS). The primary outcome was the risk of CKD, which was defined as the estimated glomerular filtration rate (eGFR) < 60 mL/min/1.73 m^2^. Multivariable logistic regression model was used to examine the relationship of serum Mg concentrations with the risk of CKD.

**Results:**

Included were 8,277 individuals, with an overall CKD prevalence of 11.8% (*n* = 977). Compared with the first quartile of serum Mg, the multivariable-adjusted odds ratios (ORs) and 95% confidence intervals (CIs) for participants in the second, third, and fourth quartiles of serum Mg were 0.74 (0.58, 0.93), 0.87 (0.69, 1.11) and 1.29 (1.03, 1.61), respectively. Similar results were observed in our several sensitivity analyses. Restricted cubic spline analysis demonstrated a nonlinear (similar “J”-shaped) association between serum Mg concentrations and the risk of CKD (*P*_nonlinearity_ <0.001), with a threshold at around a serum Mg value of 2.2 mg/dL.

**Conclusions:**

Our results suggested a similar “J”-shaped association between serum Mg concentration and the risk of CKD among Chinese adults. Further large prospective studies are needed to verify these findings.

**Supplementary Information:**

The online version contains supplementary material available at 10.1186/s12889-023-17615-2.

## Introduction

Chronic kidney disease (CKD) is an increasing and leading public health challenge worldwide, as the primary contributor to global mortality and morbidity [1]. Nearly 10% of adults worldwide suffer from CKD, resulting in nearly 35.0 million years of healthy life lost and 1.2 million deaths every year [2]. Therefore, it is of vital importance to recognize relevant and adaptable risk factors for the delay or prevention of CKD.

Magnesium (Mg), as the second most important intracellular positive ion and the fourth abundant cation in the human body [3, 4], has been linked to many diseases, including hypertension [5], cardiovascular disease [6], diabetes [7], anemia [8], as well as CKD [9]. Several epidemiologic studies have been conducted to dissect the connection between serum Mg status and CKD. For instance, the ARIC study suggested that lower serum Mg concentrations were independently linked to greater risks of CKD and end-stage renal disease (ESRD) among 13,226 middle-aged participants [10]. In a cohort study involving 1,650 Western European CKD cases, Van Laecke et al. reported that lower serum Mg concentrations were linked to a more rapid decline in kidney function [11]. The HANDLS study found a higher risk of fast renal function deterioration among 1,252 African-American and Caucasian participants with reduced dietary Mg consumption [12]. Although the above-mentioned research has revealed that serum Mg is negatively correlated with the occurrence and prognosis of CKD, these studies are limited to European and American populations. In addition, Azem et al. [13] revealed that either hyper- or hypo-magnesemia was substantially linked to a growing risk of CKD progression among 10,568 patients with CKD stage 3 and 4. Ortega et al. [14] found that serum Mg was not a reliable indicator of cardiovascular events or overall mortality among 70 non-dialysis patients with advanced CKD. Several studies have also indicated an association between Mg status and survival in ESRD patients [15–17]. Therefore, until now, the relationships between Mg and the onset, development, and prognosis of CKD are limited, inconsistent, and not well established, particularly in the Chinese population.

In this context, we sought to explore the relationships between serum Mg status and CKD in a nationally representative sample of Chinese adults using data from the China Health and Nutrition Survey (CHNS).

## Methods

### Study population and design

The CHNS is an ongoing national longitudinal survey to investigate health and the nutritional status of Chinese residents [18]. A multistage random clustering process was utilized to sample 288 communities in 12 provinces of China by 2011 (i.e., Beijing, Heilongjiang, Liaoning, Henan, Shandong, Shanghai, Jiangsu, Hubei, Guangxi, Hunan, Chongqing, and Guizhou) [19], which were stratified by income using State Statistical Office definitions. All participants voluntarily signed informed consent and this study was supported by the institutional review board from the Chinese Center for Disease Control and Prevention and the University of North Carolina at Chapel Hill. We followed the Strengthening the Reporting of Observational Studies in Epidemiology (STROBE) guideline in conducting and reporting the study [20]. The inclusion criterion was participants who had blood sample collection in the wave of 2009, and the exclusion criterion was individuals who were aged < 18 years old and had missing information. Finally, a total of 8,277 participants from the CHNS 2009 were eligible for inclusion.

### Laboratory measurements

Fasting blood samples were collected in local laboratories according to consensus guidelines with strict quality control, then transported to a national central lab in Beijing for future testing [18]. Serum Mg and creatinine were analyzed using the Xylidyl blue colorimetric method (Randox, UK) and a standard Picric acid method (Randox, UK), respectively. The measured serum Mg concentrations were substituted with the mean concentration ± 3×standard deviation for those with measured concentrations higher/lower than this value [21]. Serum high-density lipoprotein cholesterol (HDL-C) and total cholesterol (TC) were measured using the enzymatic method (Kyowa, Japan) and the cholesterol oxidase-phenol plus aminophenazone method (CHOD-PAP; Randox, UK), respectively. Serum triacylglycerol (TG) was detected using the glycerol phosphate oxidase phenol 4-aminoantipyrine peroxidase (GPO-PAP) method (Kyowa, Japan). Blood hemoglobin (Hb) was examined using the Coulter volume scatter conductivity (VCS) hematology analyzer (Beckman Coulter, USA).

### Ascertainment of CKD

The primary outcome of interest in the current study, CKD, was defined by an eGFR < 60 mL/min/1.73 m^2^ [22, 23]. The eGFR value was calculated from age (in years), sex, and serum creatinine (in mg/dL), using the Chronic Kidney Disease Epidemiology Collaboration (CKD-EPI) equation [24]. Moreover, the Modification of Diet in Renal Disease (MDRD) equation was also adopted in our sensitivity analysis [25].

### Assessment of covariates

Information on age, sex, residential place, education level, self-reported diagnosed fracture/diabetes/hypertension, and lifestyles (e.g., smoking, and drinking) were obtained from a face-to-face interview. We divide the subject’s educational level into 4 categories (no formal education, primary school, middle school, high school or higher) [26, 27]. Individual diet was repeatedly evaluated by a household food inventory survey, and 24-h dietary recalls on three consecutive days were performed to assess individual diets [28]. Dietary nutrient intakes (e.g., caloric, carbohydrate, and protein) were estimated based on the compositions in the China food composition tables (FCTs) and individual dietary consumption data [29]. Height and weight were measured using the standard scheme by trained examiners. Body mass index (BMI) was calculated as weight (kg) divided by the square of height (m). BMI cut-off points for overweight and obesity were 24.0 kg/m^2^ and 28.0 kg/m^2^ [30] for Chinese adults, respectively.

### Statistical analysis

Participants were classified according to the quartiles of serum Mg concentrations (Q1: below 2.16 mg/dL, Q2: 2.16–2.28 mg/dL, Q3: 2.28–2.43 mg/dL, and Q4: above 2.43 mg/dL). Differences between serum Mg quartile groups in socio-demographic and clinical characteristics were evaluated by the ANOVA or Kruskal-Wallis test for continuous variables and the Chi-square test for categorical variables, respectively.

Based on previous studies [31–33], to calculate the odds ratios (ORs) and 95% confidence intervals (CIs) of CKD based on quartiles of serum Mg concentrations, 3 multivariable logistic regression models were constructed. Model 1 was a crude model. In model 2, we made age (years), sex (male or female), BMI (< 18.5, 18.5–23.9, 24.0-27.9, or ≥ 28.0 kg/m^2^), residential place (rural or urban), educational level (no formal education, primary school, middle school, high school or higher), smoking (yes or no), alcohol consumption (yes or no), self-reported diagnosed hypertension, diabetes, or fracture (yes or no), and biochemical indicators (i.e., TC, HDL-C, TG, and Hb) adjustments. In model 3, we further adjusted for dietary consumption (i.e., protein, carbohydrate, and calorie intake), because of the collinearity problem, we did not adjust the variable of fat intake. Tests for trends across Mg quartiles were assessed by including the median values within each group as a continuous variable in separate models [34].

The dose-response relationship of serum Mg with CKD was tested by a 3-knots (i.e., the 5th, 50th, and 95th percentile) restricted cubic spline regression models. Stratified analyses were performed by age (≤ 60 and > 60 years), sex (male and female), BMI (< 18.5, 18.5–23.9, 24.0-27.9, and ≥ 28.0 kg/m^2^), residential place (rural and urban), educational level (no formal education, primary school, middle school, high school or higher), smoking status (no and yes), alcohol status (no and yes), self-reported diagnosed hypertension, diabetes, and fracture (no and yes), and the significance of interaction was evaluated by the likelihood ratio test.

In order to verify that our findings are reliable, the relationship between quartiles of serum Mg and eGFR calculated from CKD-EPI was analyzed using linear regression models. Besides, we utilized the modified MDRD equation to calculate eGFR, then repeated the analyses between quartiles of serum Mg and incident CKD and eGFR. All statistical analyses were conducted using Stata 15.0 (StataCorp, College Station, Texas) software, and *P* < 0.05 (two-sided) was regarded as statistically significant.

## Results

### Study participants and baseline characteristics

**Fig. 1:**
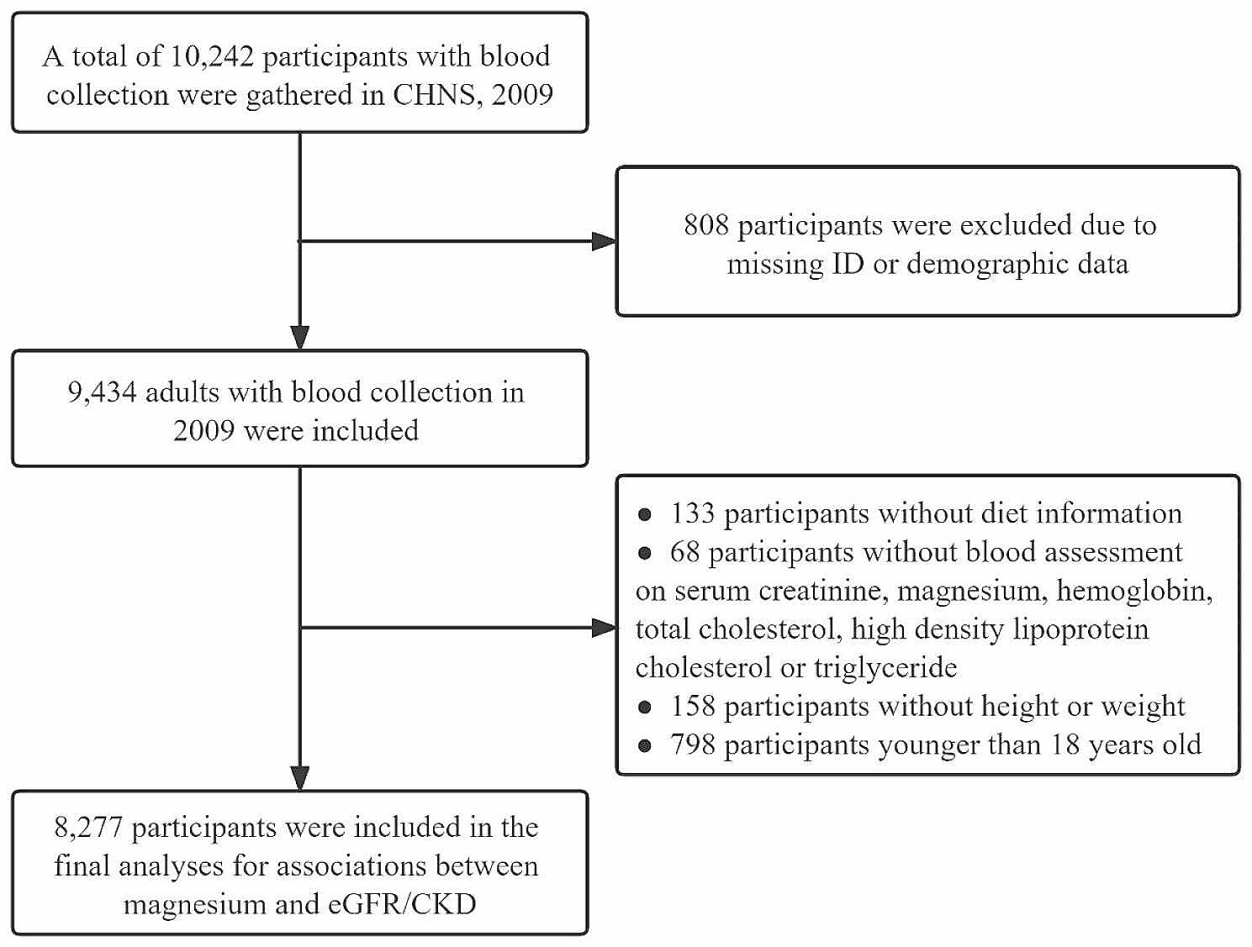
Flowchart of participant selection. CHNS, China Health and Nutrition Survey; CKD, Chronic kidney disease; eGFR, estimated glomerular filtration rate.

A total of 10,242 participants were identified from the 2009 wave of CHNS, 9,434 of whom had blood sample collection. After excluding those who were aged < 18 years old (*n* = 798), and had missing information on diet (*n* = 133), height or weight (*n* = 158), and the other biomarkers (*n* = 68). Finally, the current analysis comprised 8,277 people altogether. Details regarding the participant’s selection are shown in Fig. 1. Table 1 presents the participants’ characteristics. The quartile of serum Mg is as follows, Q1: below 2.16 mg/dL (0.90 mmol/L), Q2: 2.16–2.28 mg/dL (0.90–0.95 mmol/L), Q3: 2.28–2.43 mg/dL (0.95–1.01 mmol/L), and Q4: above 2.43 mg/dL (1.01mmol/L). The average age of the 8,277 participants in the current study was 51.3 years, 3,865 (46.7%) of them were men, and 977 (11.8%) of them were diagnosed with CKD. Subjects who had higher Mg concentrations were more likely to be older, male, lived in urban, smokers, drinkers, and obese; and had higher educational levels and a history of self-reported diagnosed hypertension. Moreover, different concentrations of serum Mg were also accompanied by significant differences in various biochemical indicators (i.e., TC, HDL-C, TG, and Hb) and dietary intake (i.e., caloric, carbohydrate, and protein) (*P* < 0.05).

**Table 1:** Characteristics of the study population according to serum magnesium concentrations in CHNS 2009.^1^

Characteristics	Magnesium						P-value
	Total		Q1	Q2	Q3	Q4	
Overall	8,277		2,091	2,308	1,884	1,994	
Age (years)	50.9 ± 15.2		49.5 ± 15.6	49.8 ± 14.7	51.8 ± 15.2	52.6 ± 14.5	**< 0.001** ^a^
**Gender**							**< 0.001** ^b^
Male	3,865 (46.7)		861(41.2)	1,046 (45.3)	904 (48.0)	1,054 (52.9)	
Female	4,412 (53.3)		1,230 (58.8)	1,262 (54.7)	980 (52.0)	940 (47.1)	
**Region**							**< 0.001** ^b^
Rural	5,757 (69.6)		1,505 (72.0)	1,637 (70.9)	1,291 (68.5)	1,324 (66.4)	
Urban	2,520 (30.5)		586 (28.0)	671 (29.1)	593 (31.5)	670 (33.6)	
**Educational level**							**< 0.001** ^b^
No formal education	1,956 (23.6)		485 (23.2)	485 (21.0)	461 (24.5)	525 (26.3)	
Primary school	1,638 (19.8)		425 (20.3)	455 (19.7)	391 (20.8)	367 (18.4)	
Middle school	2,735 (33.0)		718 (34.3)	799 (34.6)	613 (32.5)	605 (30.3)	
High school or higher	1,948 (23.5)		463 (22.1)	569 (24.7)	419 (22.2)	497 (24.9)	
**BMI (kg/m** ^**2**^ **)**							**< 0.001** ^b^
< 18.5	520 (6.3)		150 (7.2)	153 (6.6)	120 (6.4)	97 (4.9)	
18.5 ~ 23.9	4,430 (53.5)		1,215 (58.1)	1,238 (53.6)	978 (51.9)	999 (50.1)	
24.0 ~ 27.9	2,513 (30.4)		543 (26.0)	705 (30.6)	591 (31.4)	674 (33.8)	
≥ 28.0	814 (9.8)		183 (8.8)	212 (9.2)	195 (10.4)	224 (11.2)	
**Alcohol consumption**							**< 0.001** ^b^
No	5,584 (67.5)	1,482 (70.9)		1,574 (68.2)	1,267 (67.3)	1,261 (63.2)	
Yes	2,693 (32.5)	609 (29.1)		734 (31.8)	617 (32.8)	733 (36.8)	
**Smoking status**							**< 0.001** ^b^
No	5,710 (69.0)	1,516 (72.5)		1,629 (70.6)	1,258 (66.8)	1,307 (65.6)	
Yes	2,567 (31.0)	575 (27.5)		679 (29.4)	626 (33.2)	687 (34.5)	
**Hypertension**							**< 0.001** ^b^
No	7,182 (86.8)	1,846 (88.3)		2,046 (88.7)	1,624 (86.2)	1,666 (83.6)	
Yes	1,095 (13.2)	245 (11.7)		262 (11.4)	260 (13.8)	328 (16.5)	
**Diabetes**							0.134 ^b^
No	8,034 (97.1)	2,014 (96.3)		2,248 (97.4)	1,832 (97.2)	1,940 (97.3)	
Yes	243 (2.9)	77 (3.7)		60 (2.6)	52 (2.8)	54 (2.7)	
**Fracture**							**0.008** ^b^
No	7,871 (95.1)	2,004 (95.8)		2,174 (94.2)	1,779 (94.4)	1,914 (96.0)	
Yes	406 (4.9)	87 (4.2)		134 (5.8)	105 (5.6)	80 (4.0)	
Creatinine (µmol/L)	87.5 ± 23.4	85.4 ± 19.8		85.2 ± 15.2	88.0 ± 19.7	91.8 ± 34.6	**< 0.001** ^a^
HDL-C (mmol/L)	1.4 ± 0.5	1.5 ± 0.4		1.4 ± 0.4	1.4 ± 0.5	1.4 ± 0.6	**< 0.001** ^a^
TC (mmol/L)	4.9 ± 1.0	4.7 ± 1.0		4.8 ± 1.1	4.9 ± 1.0	5.1 ± 1.1	**< 0.001** ^a^
TG (mmol/L)	1.6 ± 1.4	1.3 ± 0.8		1.5 ± 1.0	1.6 ± 1.2	2.0 ± 2.1	**< 0.001** ^a^
Hemoglobin (g/L)	141.3 ± 20.7	136.1 ± 20.6		141.1 ± 20.4	142.7 ± 20.0	145.5 ± 20.6	**< 0.001** ^c^
Caloric intake (kcal)	2,137.3 ± 663.5	2,102.1 ± 678.6		2,140.9 ± 650.9	2,163.4 ± 665.6	2,145.2 ± 659.0	**0.026** ^a^
Carbohydrate intake (g)	294.6 ± 101.8	286.3 ± 99.7		297.5 ± 103.7	298.8 ± 102.2	296.0 ± 101.0	**0.002** ^a^
Protein intake (g)	65.88 ± 23.0	63.6 ± 22.3		65.9 ± 23.0	66.9 ± 23.3	67.4 ± 23.2	**< 0.001** ^c^

^1^ The statistical data are shown as means (SDs) for continuous variables and counts (percentages) for categorical variables. ^a^ Kruskal-Wallis test; ^b^ Chi-squared test; ^c^ ANOVA. BMI, body mass index; CHNS, China Health and Nutrition Survey; HDL-C, high-density lipoprotein cholesterol; Q, quartile; SD, standard deviation; TC, total cholesterol; TG, triacylglycerol.

### Association between serum Mg and CKD

**Fig. 2:**
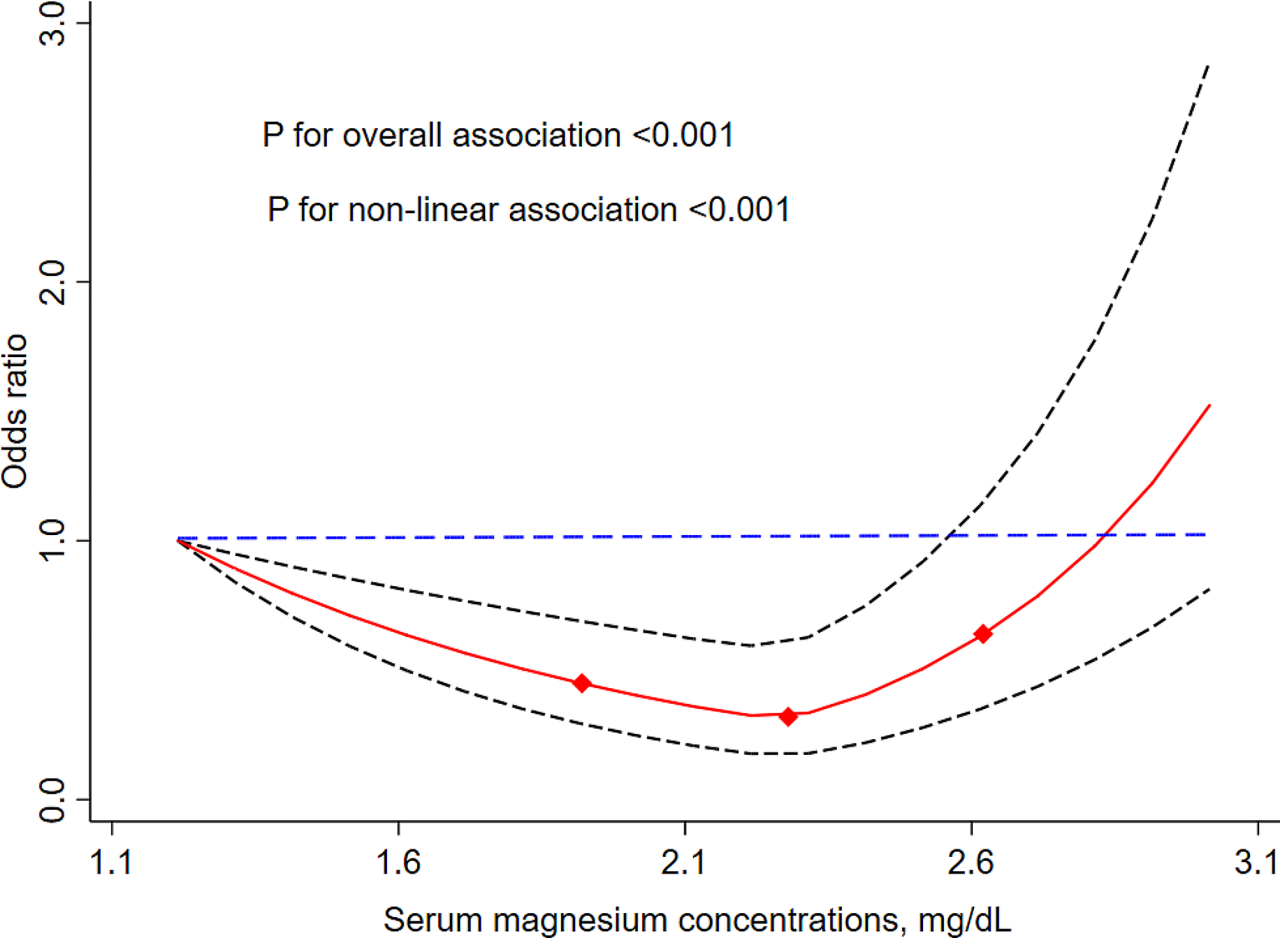
Adjusted OR (95% CIs) for chronic kidney disease by serum magnesium concentrations.^1^ ^1^Results were adjusted for age (continuous), sex (male or female), body mass index (< 18.5, 18.5–23.9, 24.0-27.9, or ≥ 28.0 kg/m^2^), residential place (rural or urban), educational level (no formal education, primary school, middle school, high school or higher), smoking status (yes or no), alcohol status (yes or no), comorbidities (i.e., hypertension, diabetes, and fracture), biochemical indicators (i.e., total cholesterol, high density lipoprotein cholesterol, triglyceride, and hemoglobin), and dietary consumptions (i.e., protein, carbohydrate, and calorie intake). The red solid lines represent the ORs, and the black dashed lines are 95% CIs. CI, Confidence interval; OR, Odds ratio

Table 2 presents the relationship between the quartiles of serum Mg concentrations and CKD in the logistic regressions. Compared with the first quartile, the crude ORs (95% CIs) for CKD associated with serum Mg concentration in the second and fourth quartiles were 0.71 (95% CI: 0.59, 0.87) and 1.38 (95% CI: 1.15, 1.65), respectively. Consistent results were observed in model 2, which was corrected for age, sex, BMI, residential place, educational level, current smoking status, current alcohol consumption, Hb, HDL-C, TC, TG, self-reported diagnosed hypertension, diabetes, and fracture. Model 3 was corrected as for model 2 and additionally adjusted for dietary consumption (i.e., protein intake, carbohydrate intake, and calorie intake), and the adjusted ORs (95% CIs) from bottom to top serum Mg categories were 1.00 (reference), 0.74 (95% CI: 0.58, 0.93), 0.87 (95% CI: 0.69, 1.10), and 1.29 (95% CI: 1.03, 1.61), respectively. The restricted cubic spline curves further demonstrated a curvilinear (similar “J”-shaped) association between serum Mg concentrations and CKD (*P*_non−linearity_ <0.001), with a threshold at around a serum Mg value of 2.2 mg/dL (Fig. 2).

**Table 2:** ORs (95% CIs) for chronic kidney disease according to serum magnesium concentrations in CHNS 2009

	Serum magnesium concentrations				*P*-trend
	Q1	Q2	Q3	Q4	
No. of cases/participants	245/2,091	200/2,308	223/1,884	309/1,994	-
Model 1	Ref	0.71 (0.59, 0.87)	1.01 (0.83, 1.23)	1.38 (1.15, 1.65)	< 0.001
Model 2	Ref	0.73 (0.57, 0.92)	0.86 (0.68, 1.09)	1.27 (1.02, 1.59)	0.012
Model 3	Ref	0.74 (0.58, 0.93)	0.87 (0.69, 1.10)	1.29 (1.03, 1.61)	0.010

Model 1: estimate without covariate;

Model 2: adjusted for age (continuous), sex (male or female), body mass index (< 18.5, 18.5–23.9, 24.0-27.9, or ≥ 28.0 kg/m^2^), residential place (rural or urban), educational level (no formal education, primary school, middle school, high school or higher), smoking status (yes or no), alcohol status (yes or no), comorbidities (i.e., hypertension, diabetes, and fracture), and biochemical indicators (i.e., total cholesterol, high-density lipoprotein cholesterol, triacylglycerol, and hemoglobin);

Model 3: further adjusted (from Model 2) for dietary consumption (i.e., protein, carbohydrate, and calorie). CHNS, China Health and Nutrition Survey; CI, Confidence interval; OR, Odds ratio; Q, quartile.

### Subgroups analyses and sensitivity analyses

**Fig. 3:**
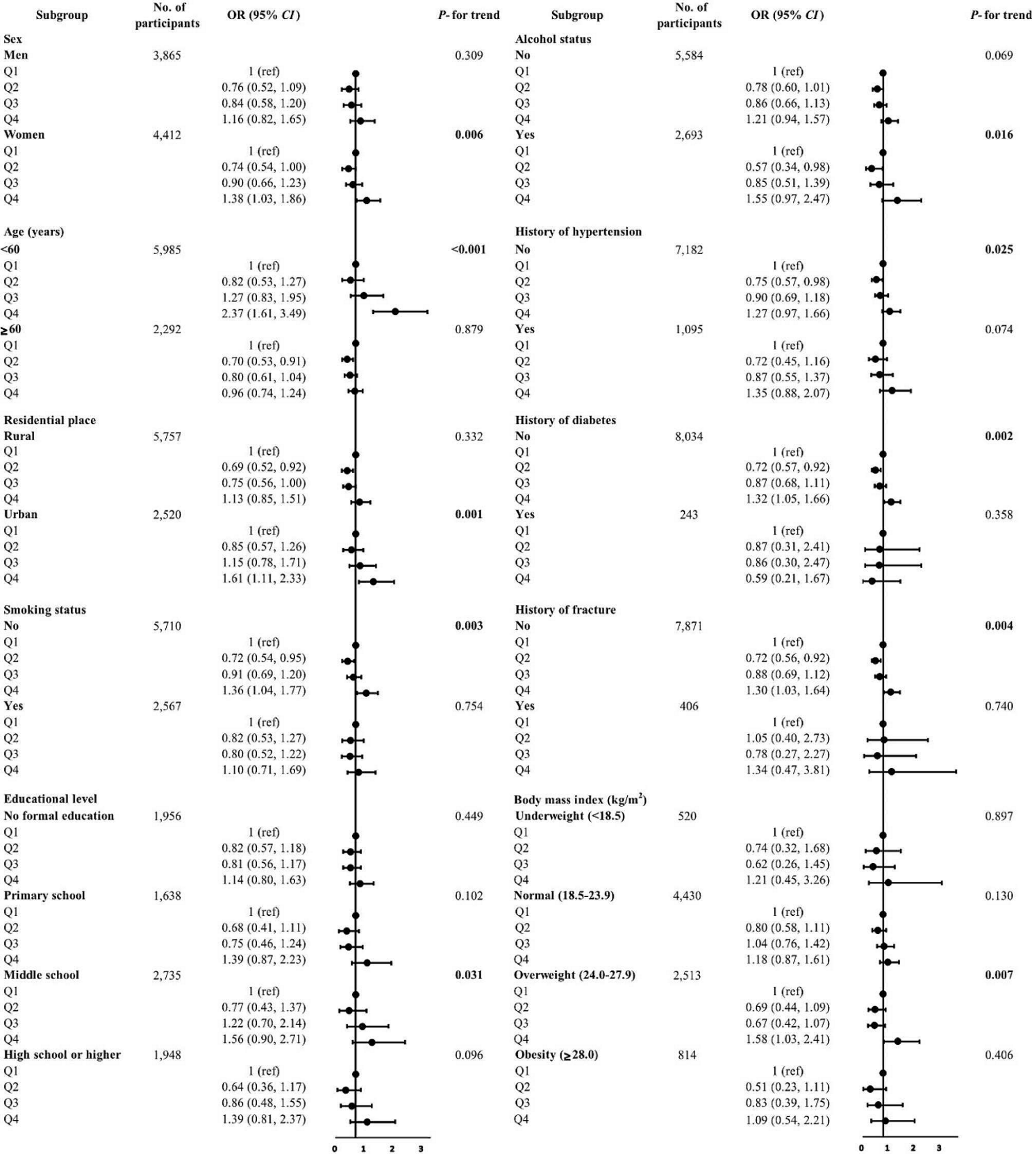
Adjusted OR (95% CIs) for chronic kidney disease by serum magnesium concentrations in subgroups analyses.^1^ ^1^Results were adjusted for age, sex, body mass index, current smoking status, alcohol drinking status, educational level, residence, history of diabetes, hypertension, and fracture, high density lipoprotein cholesterol, total cholesterol, triglyceride, hemoglobin, and dietary consumptions (i.e., protein, carbohydrate, and calorie intake), and stratified variables were not included in the relevant models. CI, Confidence interval; OR, Odds ratio; Q, quartile.

Figure 3 presents the results of stratification analyses, and similar results were obtained as analyses were stratified by age, sex, BMI, household registration, educational level, current smoking status, current alcohol consumption, self-reported diagnosed hypertension, diabetes, or fracture. No significant interaction was detected between serum Mg concentrations and these stratifying variables (all *P*_interaction_ >0.05), except for age (*P*_interaction_ <0.001).

In the full-adjusted linear regression model, the coefficients (95% CIs) of eGFR associated with the third quartile and fourth quartile of serum Mg concentration versus the first quartile was − 1.61 (95% CI: -2.38, -0.84) and − 3.62 (95% CI: -4.39, -2.84), respectively (Supplementary Table 1). These associations were still observed in the linear regression and multivariable-adjusted logistic regression analyses when we used the MDRD equation to generate eGFR (Supplementary Tables 2 and 3).

## Discussion

A similar “J”-shaped associations between serum Mg concentrations and CKD were discovered in this relatively large-scale, nationwide study among the general Chinese population. The correlation was independent of traditional risk factors, such as lifestyle factors, biochemical indicators, BMI, and dietary consumption. A variety of stratified analyses and sensitivity analyses demonstrated the robustness of our findings.

The concentrations of serum Mg (median/mean: 0.95 mmol/L) in the present study were slightly higher than those described in NHANES I study in the U.S. (median: 0.85 mmol/L) [35], and from the EPIC-Norfolk cohort study in the UK (males: 0.82 mmol/L; females: 0.80 mmol/L) [36], while were slightly lower than that reported in the rural areas of the southwest of China (median: 1.07 mmol/L) [37]. The difference in serum Mg levels among these regions may be attributed to the inclusion of age limit, dietary preference, drinking water, and race specificity [35, 38, 39].

Mg is frequently active in numerous pathophysiological changes and molecular mechanisms in the human body. As a critical cofactor, Mg could be helpful to exert biological functions in any reaction powered by adenosine triphosphate (ATP); as a phosphate binder, Mg could be helpful to decrease hyperphosphatemia and vascular calcification [40, 41]; as a calcium channel antagonist, Mg could involve in regulating various activity accompanied by intracellular calcium concentration fluxes, such as contraction of muscles, release of insulin [42]. Thus, Mg disorder, probably owing to an excessive dietary intake of Mg, pathological problems (e.g., abnormal renal wasting), or excessive use of drugs (e.g., diuretics) [43], would give rise to serious consequences or dramatic effects on metabolism. Consistent with this notion, our current study showed a similar “J”-shaped relationship between serum Mg levels and CKD among 8,277 Chinese adults, which indicated that either serum Mg deficiency or surplus might be linked to CKD. The risk of CKD development significantly decreased with the increment of serum Mg in participants when serum Mg < 2.2 mg/dL, but if serum Mg ≥ 2.2 mg/dL, the relationship between them developed in the opposite direction. Similar to our study, in 10,568 patients with CKD stage 3 and 4, Azem et al. [13] reported that either hyper- or hypo-magnesemia was significantly associated with an increased risk of CKD progression. In a cohort study with 142,555 hemodialysis patients, Sakaguchi et al. [44]. found that the risk of all-cause mortality was much higher in sextiles 1–3 and 6 of Mg concentration, which suggested that there may be an optimal concentration of Mg. Therefore, it could be speculated that the concentration of Mg should be controlled at an appropriate range, which needed to be performed and verified in future studies. In addition, we found that the association between serum Mg and CKD was not significant in older individuals who were above 60 years, perhaps since serum Mg concentrations generally decrease with age due to uneven diet, chronic diseases or side effects of medication, while the risk of developing CKD increased with age [45]，then this relationship was weakened [46].

The relevant mechanisms underlying the similar “J”-shaped relationships between serum Mg concentrations and CKD remain incompletely clear, and several possibilities could be proposed to decipher our findings. Firstly, Mg may involve the prevention of nephrocalcinosis, inhibition of phosphate-mediated apoptosis of tubular cells and calcification of renal arteries, and suppression of tubular calcium phosphate crystallization, which may protect against phosphate-induced kidney damage. In addition, tubular dysfunction and interstitial fibrosis could contribute to the loss of Mg [47, 48]. Moreover, matured calciprotein particles have the capacity to induce vascular calcification, and recent studies have shown that Mg could prevent the maturation of calciprotein particles [49], and these may be the basics that underlie the anti-calcification properties of Mg. Secondly, Mg exercises the functions of protein metabolism and energy synthesis. Therefore, Mg deficiency may induce the development of CKD by reducing protein synthesis and energy metabolism of renal reparative cells. Thirdly, the concentrations of antioxidants (e.g., selenium and vitamin C) are decreased in the context of Mg deficiency [50], which was closely related to promoting oxidative stress. In addition, related studies conducted in vitro on endothelial cells have shown that a low Mg medium promotes inflammation and oxidative stress, and induces the expression of proatherothrombotic molecules such plasminogen activator inhibitor-1 and vascular cell adhesion molecule-1 [51], and Mg deficiency was found to be linked to endothelial dysfunction [52], therefore, we reasonably speculate that Mg may have protective effects on endothelium, and Mg deficiency could be responsible for endothelial dysfunction and vascular sclerosis of renal vessels, accelerating the progression of kidney damage. Finally, previous studies also have shown that chronic inflammation is crucial to the development of CKD [53, 54], thus abnormal Mg levels (deficiencies or excesses) may aggravate inflammation-related renal injury.

The large sample size and population-based design of our study were its major strengths, allowing us to perform a series of stratifications and the results were reliable and generalizable to the general population in China. However, our research has certain limitations as well. First, we were unable to establish the causal link between serum Mg and CKD because of the cross-sectional nature of this study. It is still needed to affirm whether the reduced renal function is a crucial risk element for hypermagnesemia or not, maybe the hypermagnesemia results in the faster development of CKD or the compensatory protective elevation of serum Mg for delaying the progression of CKD. Secondly, serum Mg concentrations were assessed at a single time point, thus the measured data may not be objective to reflect the whole life-course activity, and there is no more relevant available data on Mg intake in this database, so we cannot conduct subsequent causal analyses. Thirdly, due to a lack of data on the severity of CKD, we could not determine whether these associations differed by the severity of CKD; while the prevalence of CKD in our study is comparable to that estimated in global analysis (8.0–16.0%) [55]. Finally, although we have considered several potential confounders, including various lifestyles and dietary variables, additional unmeasured confounders cannot be fully excluded.

## Conclusions

In this relatively large-scale, national study, we found a similar “J”-shaped association between serum Mg concentrations and CKD among Chinese adults. Our findings highlight the possibility of maintaining the optimal serum Mg concentrations for the management of CKD, and present the feasibility that Mg might be an intervention target for the treatment of CKD.

### Electronic supplementary material

Below is the link to the electronic supplementary material.

Supplementary Material 1

## Data Availability

Data of CHNS can be viewed and obtained from the following website: https://www.cpc.unc.edu/projects/china, and further inquiries can be directed to the corresponding authors.

## Abbreviations

BMI: Body mass index
CKD: Chronic kidney disease
eGFR: Estimated glomerular filtration rate
TC: Total cholesterol
HDL-C: High-density lipoprotein cholesterol
Mg: Magnesium
HT: Hypertension
OR: Odds ratio
