# Supplementary material for: The association between serum magnesium and chronic kidney disease in Chinese adults: a cross-sectional study
Source: BMC Public Health. 2024 Jan 15;24:187. doi: 10.1186/s12889-023-17615-2 (PMC10790542; doi:10.1186/s12889-023-17615-2)
Supplement: Supplementary file 1 — Supplementary Material 1 [file 12889_2023_17615_MOESM1_ESM.docx]

**Supplementary Table 1 |** Coefficients (95% CIs) for estimated glomerular filtration rate according to serum magnesium concentrations in CHNS 2009.

|  | **Serum magnesium concentrations** | | | | ***P-*trend** | |
| --- | --- | --- | --- | --- | --- | --- |
|  | **Q1** | **Q2** | **Q3** | **Q4** |  |  |
| No. of cases/participants | 245/2,091 | 200/2,308 | 223/1,884 | 309/1,994 | - | |
| Model 1 | Ref | 0.21 (-0.79, 1.21) | -2.90 (-3.95, -1.85) | -5.07 (-6.10, -4.04) | | <0.001 |
| Model 2 | Ref | 0.21 (-0.52, 0.94) | -1.54 (-2.31, -0.77) | -3.54 (-4.32, -2.77) | <0.001 | |
| Model 3 | Ref | 0.12 (-0.60, 0.85) | -1.61 (-2.38, -0.84) | -3.62 (-4.39, -2.84) | <0.001 | |

Model 1: estimate without covariate;

Model 2: adjusted for age (continuous), sex (male or female), body mass index (<18.5, 18.5-23.9, 24.0-27.9, or ≥28.0 kg/m^2^), residential place (rural or urban), educational level (no formal education, primary school, middle school, high school or higher), smoking status (yes or no), alcohol status (yes or no), comorbidities (i.e., hypertension, diabetes, and fracture), and biochemical indicators (i.e., total cholesterol, high-density lipoprotein cholesterol, triacylglycerol, and hemoglobin);

Model 3: further adjusted (from Model 2) for dietary consumption (i.e., protein, carbohydrate, and calorie intake). CHNS, China Health and Nutrition Survey; CI, Confidence interval; Coef, coefficient; Q, quartile.

**Supplementary Table 2 |** ORs (95% CIs) for chronic kidney disease according to serum magnesium concentrations when using the modified Modification of Diet in Renal Disease equation to estimate eGFR.^1^

|  | **Serum magnesium concentrations** | | | | ***P-*trend** |
| --- | --- | --- | --- | --- | --- |
|  | **Q1** | **Q2** | **Q3** | **Q4** |  |
| No. of cases/participants | 149/2,091 | 125/2,344 | 142/1,906 | 205/2,013 | - |
| Model 1 | Ref | 0.75 (0.58, 0.95) | 1.06 (0.84, 1.35) | 1.49 (1.20, 1.86) | <0.001 |
| Model 2 | Ref | 0.81 (0.62, 1.06) | 0.98 (0.76, 1.28) | 1.39 (1.08, 1.78) | 0.004 |
| Model 3 | Ref | 0.83 (0.63, 1.08) | 1.00 (0.77, 1.30) | 1.40 (1.09, 1.80) | 0.003 |

Model 1: estimate without covariate;

Model 2: adjusted for age (continuous), sex (male or female), body mass index (<18.5, 18.5-23.9, 24.0-27.9, or ≥28.0 kg/m^2^), residential place (rural or urban), educational level (no formal education, primary school, middle school, high school or higher), smoking status (yes or no), alcohol status (yes or no), comorbidities (i.e., hypertension, diabetes, and fracture), and biochemical indicators (i.e., total cholesterol, high-density lipoprotein cholesterol, triacylglycerol, and hemoglobin);

Model 3: further adjusted (from Model 2) for dietary consumption (i.e., protein, carbohydrate, and calorie intake). CI, Confidence interval; eGFR, estimated glomerular filtration rate; OR, Odds ratio; Q, quartile.

**Supplementary Table 3** **|** Coefficients (95% CIs) for estimated glomerular filtration rate by MDRD formula according to serum magnesium concentrations in CHNS 2009.

|  | **Serum magnesium concentrations** | | | | | ***P-*trend** | | |
| --- | --- | --- | --- | --- | --- | --- | --- | --- |
|  | **Q1** | **Q2** | **Q3** | **Q4** |  | |  |  |
| No. of cases/participants | 149/2,091 | 125/2,344 | 142/1,906 | 205/2,013 | | - | |  |
| Model 1 | Ref | 0.11 (-0.95, 1.17) | -3.06 (-4.18, -1.95) | -5.17 (-6.27, -4.07) | | <0.001 | |  |
| Model 2 | Ref | 0.18 (-0.75, 1.12) | -2.00 (-2.99, -1.01) | -4.16 (-5.16, -3.17) | | <0.001 | |  |
| Model 3 | Ref | 0.07 (-0.86, 1.01) | -2.09 (-3.08, -1.10) | -4.26 (-5.26, -3.27) | | <0.001 | |  |

Model 1: estimate without covariate;

Model 2: adjusted for age (continuous), sex (male or female), body mass index (<18.5, 18.5-23.9, 24.0-27.9, or ≥28.0 kg/m^2^), residential place (rural or urban), educational level (no formal education, primary school, middle school, high school or higher), smoking status (yes or no), alcohol status (yes or no), comorbidities (i.e., hypertension, diabetes, and fracture), and biochemical indicators (i.e., total cholesterol, high-density lipoprotein cholesterol, triacylglycerol, and hemoglobin);

Model 3: further adjusted (from Model 2) for dietary consumption (i.e., protein, carbohydrate, and calorie intake). CHNS, China Health and Nutrition Survey; CI, Confidence interval; MDRD, Modification of Diet in Renal Disease; Q, quartile
